# The recent use of *Swietenia mahagoni* (L.) Jacq. as antidiabetes type 2 phytomedicine: A systematic review

**DOI:** 10.1016/j.heliyon.2020.e03536

**Published:** 2020-03-10

**Authors:** Sukardiman, Martha Ervina

**Affiliations:** a Department of Pharmacognosy and Phytochemistry, Universitas Airlangga, Indonesia; b Doctoral Program of Pharmaceutical Sciences, Faculty of Pharmacy, Universitas Airlangga, Indonesia; c Faculty of Pharmacy Widya Mandala Catholic University, Surabaya, Indonesia

**Keywords:** Toxicology, Diabetes, Hypoglycemic, Mahogany, Meliaceae, *Swietenia* sp

## Abstract

**Background:**

Natural resources provide more efficient and safer alternatives in managing diabetes compare to the synthetic oral anti diabetes (OAD). The plants not only have hypoglycemic effect, but also prevent its complications; in which no synthetic drugs provide of both properties. Among antidiabetes plants, mahogany seed (*Swietenia macrophylla*) has been used as traditional medicine in Indonesia and India, though most popular utilized as timber wood.

**Methods:**

The present study was performed of chemotaxonomic approach to review its phytochemical and anti-diabetic properties of *Swietenia mahagoni* (L.) Jacq seed/bark/leaves. The qualitative systematic review (SR) was carried out by analysing indexed journals and peer reviewed of *Swietenia* and *Swietenia* spp from Scopus, PubMed, Medline, Google Scholar, and Research Gate. Data selection criteria are accordance to botany, phytochemistry, *in vitro*, *in vivo*, and clinical test of related subject. The keywords used for the search in the databases were *Swietenia*, *Swietenia* mahagony, diabetes, and diabetes plants.

**Results:**

*Swietenia mahagoni* (L.) Jacq. extracts have shown *in vitro*, *in vivo* and limited clinically test of its anti-diabetic properties. Ethanolic/methanolic/aqueous/petroleum/n-hexane extracts of mahagonis's seed/bark or leaves have anti-diabetic activities comparable to the synthetic drug and observed no to relatively mild toxic effect. The hypoglycemic mechanism suggested via reducing blood glucose level, restoring liver and *β*-cells islet function (might) blocking epinephrine function, inhibiting of *α*-amylase and *β*-glucosidase, antioxidant and antihiperlipidemia. Phytochemical compounds of *S. mahagoni* consist of the phenolics (flavonoids (swietemacrophyllanin, catechins and epichatechins) and tannins), triterpenoids and tetranortriterpenoids (limonoids: mahonin, secomahoganin, swietmanins, swiemahogins, swietenine and swietenolide), saponins and alkaloids which are known as anti-diabetic bioactive principles.

**Conclusion:**

*S. mahagoni* was potentially used and developed as an antidiabetes source. To use it as an antidiabetic further, more extensive clinical trials and biomarkers of active compounds determination are needed.

## 1 Introduction

Diabetes is endocrine chronic metabolic disorder in insulin production or insulin resistance. It is diagnosed with hyperglycemia and other parameters such as HbA1c. Insulin is one of among the hormones which regulate the blood glucose level (BGL). It facilitates glucose energy source consume in most of the cell and also to store as glycogen in the liver or as a fat in the tissue. Other hormones (glucagon, amylin, cortisol, epinephrine, growth hormone, glucagon-like peptide-1 and polypeptide glucose-dependent insulinotropic) are indirectly influence BGL by leaven of insulin production (Khardori, 2018). Uncontrolled diabetes hyperglycemia overtime, leads to serious impairment of vascularization of the body's systems, reduces the quality of life with disabilities to causes premature death. Micro-vascular diabetes complication damages the nerves (neuropathy), the eyes (retinopathy), the kidneys (nephropathy) and blood vessels to the heart; while macro-vascular complications cause ischemic heart disease, stroke to peripheral vascular disorder. Diabetes prevalence estimates more than two times at 2030 from 171 million people (2000) and will be seventh leading cause of death. It will be global burden for low- and middle-income countries, so diabetes was one of four targeted priorities of non-communicable diseases in the 2011 (World Health Organization, 2016).

The classifications of diabetes are important in managing diabetes and delaying its complication. Diabetes therapy objective is to sustain normal BGL with diet, physical activities, medication; blood glucose level regularly check and complications therapy. For type 2 diabetes, life style management (diet, medical nutrition, weight control, and exercises) is important, before the use of oral anti-diabetic drugs (OAD) and or insulin injections treatment (American Diabetes Association, 2017).

Though the developing of OAD has changed with new drug classes provided and the results of medication are beyond the reach of medical therapy previously; still OADs have limitation on its side or adverse reaction effects. Some of OAD may lose its efficacy in a significant percentage of patients (Pandey et al., 2011). OAD's cause weakness and fatigue to lactic acidosis, weight gain of hyper-insulinemia, nausea, vomiting, diarrhea pancreatitis, to thyroid tumor inhibitor (Khardori, 2018). Regardless the terms of effectiveness and safety; the threat of diabetes to global endemic have encouraged source finding to alternative or complementary in diabetes therapy.

Plants provide great alternatives to manage diabetes. It was used in many developing countries with natural diversity resources. The plants are not only hypoglycemic or insulin mimetic, but also preventing the complications; which no synthetic drug provide both properties. Some have been shown in *ß*-cells regeneration function and delaying the insulin resistance, while others have antioxidant and cholesterol lowering activities. More than 1200 plants were found in ethno-pharmacological surveys for blood sugar lowering properties (Pandey et al., 2011). Here some of the plants with in the list: *Aloe vera* and Allium spp (Liliaceae), Bilberry (Moraceae), Bitter melon/*Momordica charantia* (Cucurbitaceae) (Perumal et al., 2015; Meles et al., 2019), Cinnamon spp (Lauraceae), Ginger/*Zingiber officinale* (Zingiberaceae), Fenugreek/*Trigonella foenum graecum* (Fabaceae), Ochra/*Abelmoschus esculenthus* (Malvaceae) and *Brassica juncea* (Cruciferae.), *Gymnema sylvestre* (Apocynaceae) and *Azadirachta indica* (Meliaceae) (Arumugam et al., 2013; Patel et al., 2012), *Moringa oleifera* (Amelia et al., 2018), *Garcinia mangostana* (Ansori et al., 2019; Husen et al., 2017), *Tinospora crispa* (Arundina et al., 2017; Roestamadji et al., 2017), *Cassia fistula* (Noorhajati et al., 2012). Even some mangrove plants were used for diabetes such as *Acanthus ilicifolius, Hisbiscus tiliaceus, Ipomoea pes-capre* (Purnobasuki, 2004) and also bee related product propolis (bee glue) (Trusheva et al., 2013). The plants are used by empirical base and some are supported by preclinical to clinical studies.

Among the plants previously described, mahogany seed (*Swietenia macrophylla*) has been used as traditional medicine in Indonesia (Kadota et al., 1990) and India (Arumugam et al., 2013; Patel et al., 2012; De et al., 2011). The present study was performed chemotaxonomic approach to review phytochemical and anti-diabetic properties of *Swietenia mahagoni* (L.) Jacq.

## 2 *Swietenia mahagoni* botanical view

*Swietenia* is genus of chinaberry family (Meliaceae). It was brought into some Asian countries to Bolivia from Caribbean, Mexico and Southern to Central America. Three species based of geographically separated are known; which are *Swietenia mahagoni* (L.) Jacq (West Indian mahagoni); *Swietenia humilis* Zucc (Pacific Coast *mahagoni*); and *Swietenia macrophylla* King (Honduran mahagoni) (Orwa et al., 2009).

*S. mahagoni* Jacq. is a small to medium deciduous tree (up to 30 m high), in spherical crown, short and buttressing base with diameter up to 1 m. The bark is smoothing grey and turning to scaly dark reddish-brown with many heavy branches and dense shade. The leaves phylotaxis are even and pinnate, size of 10–18 cm length, with 4–10 pairs of leaflets. The leaves are dark shiny green, lance-shaped of 2.5–5 cm and 0.7–2 cm. Its flowers are unisexual, greenish-yellow, panicles axillary and glabrous appearance. The seed capsules are green to light brown varies, upright stands, its size is about 6–10 cm and 4–5 cm of diameter. The splitting upward valves produced about 20 flat, brown-winged seeds, 4–6 cm of each. Flowering and fruiting of *Swietenia* are regular and annual. Developing of flower to mature fruit is of 8–10 months. Insect pollination and hybridization among the species is frequent, especially with *S. macrophylla*. *S. mahagoni* grows at a moderate rate and its wood mostly used (Orwa et al., 2009) (Divya et al., 2012).

## 3 Ethnomedicinal and pharmacological used of *Swietenia* spp.

*S. mahagoni* is used as medicinal plants in India (Ayurvedic system), some African countries, also in Indonesia (Patel et al., 2012.) and Malaysia (Hashim et al., 2013). Traditionally it uses for malaria, hypertension, diabetes and diarrhea, as antipyretic, as bitter tonic and astringent (Bourdy et al., 2000). Table 1 presents plant part and traditional technique on the use of *Swietenia* spp. Pharmacological activities of *S. mahagoni* are antimicrobial, anti-inflammatory, hepatoprotective, antidiarrheal, antiulcer, depressant, anticonvulsant and neuropharmacological, anti-diabetic, anti-HIV, immunomodulator, insect repellent and larvicidal, antifungal, antioxidant, analgesic, platelet aggregation inhibitors, antimutagenic and anticancer (Divya et al., 2012; Hashim et al., 2013; Bourdy et al., 2000; Moghadamtousi et al., 2013; Naveen et al., 2014; Bhurat et al., 2011).

**Table 1:** Traditional use of *Swietenia* spp.

Region	Plants part and method to use
Malaysia	Raw seeds of *S. macrophylla* are chewed/pounded to treat hypertension, diabetes and relieve pain. Decoction of chrushed seed for treating skin ailments and wounds (Kadota et al., 1990).
Amazonian Bolivian	Mashed seed of *S. macrophylla* in water is internal used for abortion, while external use for leishmaniasis (Bourdy et al., 2000)
India	- The bark decoction of *S. mahagoni* is orally taken for diarrhoea, dysentery and haemostyptic since its vitamins and iron contents. It also serves antipyretic and tonic, increasing appetite, restoring in tuberculosis, treating anaemia, fever and toothache (Khare, 2007) - Its leaf decoction is used against nerve ailments, while the leaf or root poultice for bleeding (Anonymous, 1986) - The seed infusion against chest pain (Miroslav, 2005). - The aqueous extract of *S. mahagoni* seed and bark for psoriasis, diabetes, diarrhea and as an antiseptic in cuts and wounds in East Medinipur (West Bengal), Balasore (Orissa) (Haldar et al.*,* 2011).
Indonesia	The crushed of *S mahagoni* seed water decoction for hypertension, controlling blood glucose, treating constipation, and menstrual pain, eczema and rheumatism, improving fertility and appetizing, relieving fever and cold. It also uses for powder external use as insect repellant (Mardisiswojo and Radjakmangunsudarso, 1965; Kadota et al., 1990).

## 4 Phytochemical of *Swietenia mahagoni* (L.). Jacq

Phytochemicals content of *S. mahagoni* are phospholipid, alkaloids, phenols, flavonoids, antraquinones, saponins, terpenoids, cardiac glycosides, volatile oils and long chain unsaturated acid. The contents are including 45 limonoids such as swietenolide, swiemahogins A and B, 2-hydroxy-3-*O*-tigloylswietenolide, andirobin, mexicanolide, gendunin and phragmalin, triterpens, tetranortriterpenes, swietenine dimeric triterpmahonienoid and chlorogenic acid (Patel et al., 2012; Kadota et al., 1990), swietenine acetate; 3,6-di-*0*-acetylswietenolide, 3*-0*-tigloylswietenolide, 6-acetyl-3-tigloylswietenolide, 2-*α*-hydroxymexioanolide, 6-acetylswietenine (Kadota et al., 1990; Naveen et al., 2014; Bhurat et al., 2011). Mostafa et al. (2011) found that *S. mahagoni* seed oil which has bitter taste, moderate drying oil and high content on unsaturated fatty acid, considered as useful source for soap and dying industries. The unsaturated fatty acid contents as listed in Table 2. Moghadamtousi et al. (2013) reviewed the isolated phytochemicals content from seed, bark, twig, leaves and stem of *S. macrophylla*. Most of them are limonoids (81.91%), polyphenolics (4.26%), steroids (4.26%), essential oils (6.38%), fatty acids (1.06%), coumarin (1.06%) and lignan (1.06%).

**Table 2:** Some of phytochemicals of *Swietenia* spp.

Parts of the plant	Contents (%)	References
Seed of *S. mahagoni*	Secomahoganin (0.0014), swietemahonin F (0.0036), swietemahonolide (0.0009), swietemahonin C (0.0012), 3-*O*-tiogloyl-6-*O*-acetylswietenolide (0.0003), 6-acetoxygedunin (0.0006), 7-deacetoxy-7-oxogedunin (0.0007), methyl angolensate (0.0014), swietemahonin B (0.0023)	Kadota et al. (1990)
Seed *S. mahagoni*	Swietemahonin A (0.0002), D (0.0024), E (0.0003), and G (0.0040) and 3-*O*-acetylswietenolide (0.0010) and 6-*O*-acetylswietenolide (0.0015)	Ekimoto et al. (1991)
Seed *S. mahagoni*	Swiemahogins A (0.0010) and B (0.0001)	Chen et al. (2007)
Fruits of *S. mahagoni*	Mexicanolide-type limonoids, swietmanins A (0.00005), B (0.0033), C (0.00008), D (0.00005), E (0.00008), F (0.0003), G (0.0002), H (0.0003), I (0.0003) and J (0.00005). (2-Hydroxy-3-*O*-isobutyrylproceranolide (0.0005), 2-Hydroxy-3-*O*-benzoylproceranolide (0.00008), andirobin-type limonoid, swietmanin J, mexicanolide (0.0006), 3,8-hemiketalcarapin (0.0003), 8R-hydroxycarapin (0.00025), and fissinolide (0.0002); khivorin (0.0004), and 2-hydroxyfissinolide (0.0004), 2,3-dihydroxy-3- deoxymexicanolide (0.0004) and 2-hydroxyfissinolid (0.0167), methyl angolensate (0.0002), 6-hydroxyangolensate (0.0005), and 3-deacetylkhivorin (0.0002), 3,7-dideacetylkhivorin (0.0003), 1,3,7-trideacetylkhivorin (0.0003), and 7-deacetoxy-7-oxogedunin (0.0002), and 7-deacetylkhivorin (0.0004), and 1-deacetylkhivorin (0.0005), 2-hydroxy-6-deoxyswietenolide tiglate (0.00025) and seneganolide A (0.0002)	Lin et al. (2011)
Seed *S. mahagoni*	Moisture (14.37), minerals (16.36), fats (19.42), crude fiber 19.60), protein (8.76) and carbohydrate (21.49) Fatty acid of the seed's oil: -linoleic acid (26.00), elaidic acid (24.39), stearic acid (14.32), palmitic acid (12.97), 10-methyl-10-nonadecanol (5.24), ecosanoic acid (2.48), 3-heptyne-2,5-diol-6-methyl-5-(1-methylethyl) (2.03) octadecanoic acid, 9,10,12-trimethoxy (1.90); 1,3-dioxalane-4-ethyl-4-methyl-2-pentadecyl (1.89) and 2-furapentanoic acid (1.03).	Mostafa et al. (2011)
Bark *S. macrophylla*	Swietemacrophyllanin (2R∗, 3S^∗^, 7″R∗)-catechin-8,7″-7,2″-epoxy-(methyl-4″,5″-dihydroxyphenylpropanoate) (0.0002), catechin (2,3-trans-5,7,3′,4′-tetrahydroxyflavan-3-ol) (0.0004), and epicatechin (2,3-cis-5,7,3′,4′-tetrahydroxyflavan-3-ol) (0.0003)	Falah et al. (2008)
Seed *S. macrophylla*	Swietemacrophin (0.0012). *humilinolide F* (0.0011),. *3,6-O,O-diacetylswietenolide* (0.0015), *3-O-tigloylswietenolide* (0.0012), *swietemahonin E* (0.0014). *swietenine* (0.0051)	Chen et al. (2015)
Twigs of *S. macrophylla*	Swietenitins N (0.0002), O (0.0002), P (0.00005), Q (0.00003), R (0.00005), S (0.00005), T (0.00010), U (0.00003), V (0.00005), W (0.00002), X (0.00002), epoxyfebrinin B (0.00005)	Lin et al. (2011)

**Figure 1:**
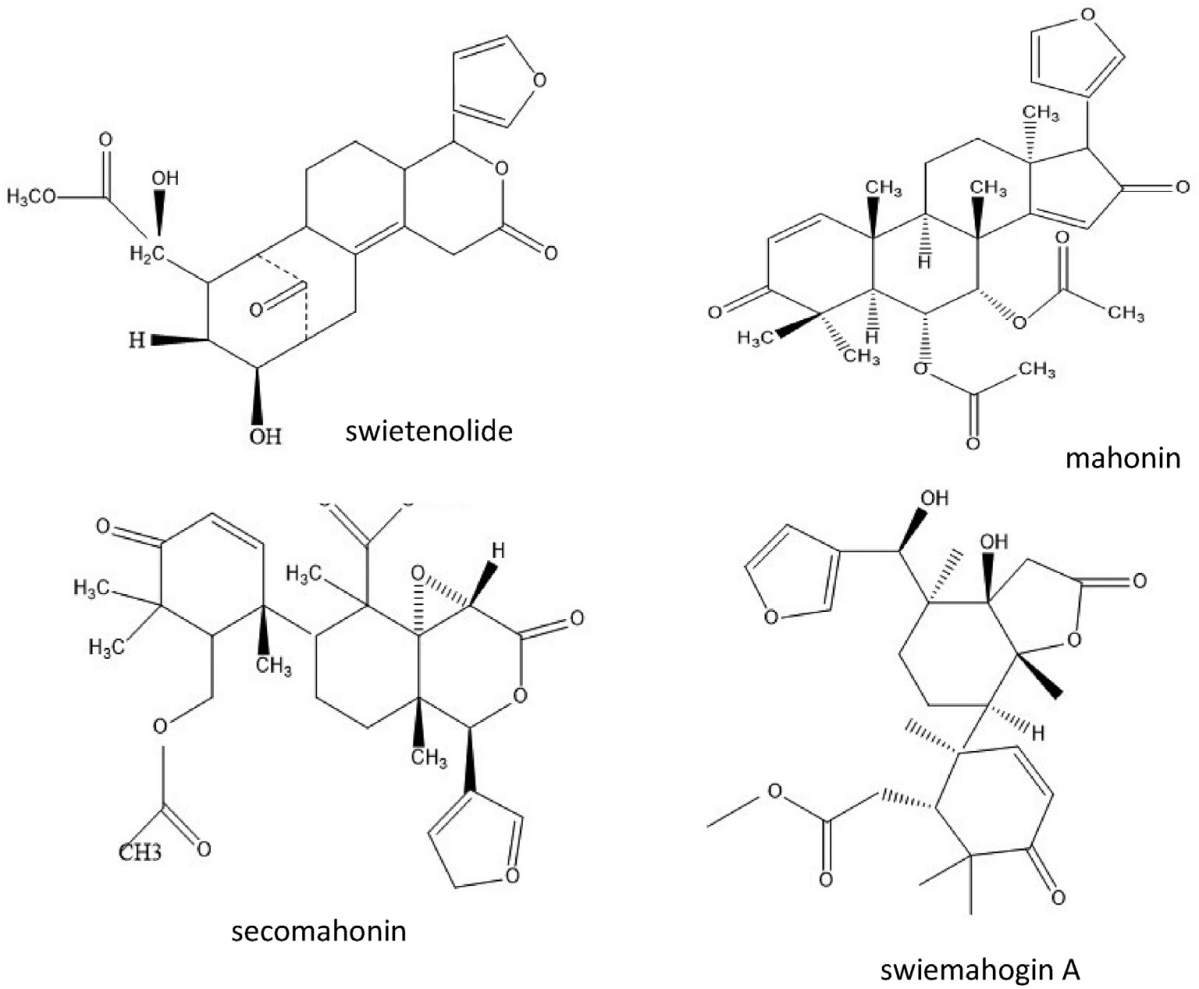
Some of phytochemical content of *S. mahagoni* L.

The leaves of *S. mahagoni* is contain tetracyclic triterpene (cyclomahogenol) (Chakraborty and Basak, 1971). The twigs and leaves produced limonoids, swiemahogins A (Figure 1) and B, which first andirobin and phragmalin types of limonoids (Chen et al., 2007). While Lin et al. (2011) isolated eleven swietenitins N to X limonoids and known epoxyfebrinin B compound from the twigs of *S. macrophylla* with percentage as written in Table 2.

The main phytoconstituents of methanolic and water extract of mahagony seeds are tannins, alkaloids, saponins and terpenoids (Hajra et al., 2011), anthraquinones, cardiac glycosides and volatile oils (Sahgal et al., 2009). Limonoids swietenolide (Figure 1) and 2-hydroxy-3-*O*-tigloylswietenolide were isolated from the methanolic extracts of the seed; which have antimicrobial activity (Rahman et al., 2009). Meanwhile in the ether extract was isolated 28 tetranotriterpenoids; which related to swietenine and swietenolide (Kadota et al., 1990). Some of these compounds are listed in Table 2. Among those substances, swietemahonins A, D, E and G and 3-*O*-acetyl-swietenolide and 6-*O*-acetyl-swietenolide inhibited platelet activating factor (PAF), thus induced platelet aggregation activity (Ekimoto et al., 1991). Another two tetranotriterpenoids of the seed were mahonin and secomahoganin (Figure 1).

Thirty compounds were isolated from the *S. mahagoni* fruits, which consisted of eleven mexicanolide-type limonoids (swietmanins A to I, 2-hydroxy-3-*O*-isobutyrylproceranolide, 2-hydroxy-3-*O*-benzoylproceranolide), and andirobin-type limonoid, swietmanin J, and 19 known compounds (Lin et al., 2009). Falah et al. (2008) found no different chemical constituents of *Swietenia*'s bark on methanol to water extract. Some phytochemical compounds such as flavonoids, triterpenoids, alkaloids and phenolics are known as bioactive antidiabetic principles (Nagappa et al., 2003; Battu et al., 2007). Furthermore, flavonoids have known as antioxidant class compound (Falah et al., 2010), which have advantage synergistic role for anti-diabetic.

Other result was reported on the phytochemical qualities parameters of *S. mahagoni* bark. It described the total and sulphated ash, water soluble and total acid insoluble ash content of the bark to be 22.0%, 14.5%, 1.4% and 0.6% respectively (Sukardiman et al., 2016); and tannin content (15.0%) (Divya et al., 2012). Sukardiman et al. (2016) was report on the quality of Indonesian dried Mahagony seed. The physical characteristic of the bark was described as flat and corrugated shaped, with 3–5 cm to 2–3 cm of the size; while the seed microscopic was observed of testa, endosperm, schlereids, essential oil and amylum fragments. It was also determined its phytochemical parameters and stigmasterol content of dried part and ethanolic extract of Mahagony seed. The study concluded its quality appropriate with Indonesia Herbal Pharmacopeia. Table 2 presents percentage of the phytochemicals isolated from *S. mahagoni* and *S. macrophylla*.

## 5 *Swietenia* spp. for diabetes

*Swietenia* seed and bark empirical most used for diabetes. Following presents scientific development on the antidiabetes research on *Swietenia* spp. *In vitro* antidiabetic potency was obtained by Hajra et al. (2011), who found ethanolic extract of *S. mahagoni* seed (EEMS) inhibited α-amylase. Furthermore Wresdiyati et al., 2015 observed its aqueous and ethanol extract (maceration and reflux methods extraction) at doses 100–500 mg/kgBW, have α-glucosidase inhibition and *in vivo* hypoglycemic activities. The test obtained best α-glucosidase inhibition was produced by ethanolic extract maceration methods. They were also showed *in vivo* antidiabetes effects.

Raja (1990) did *in vivo* treatment of ethanolic extract of mahagony seed (91,0 mg/dl) and found that it decreased BGL of the rat at 180 min Li et al. (2005) was shown *Swietenia mahagoni* extract at the dose of 1000 mg/kg (*Sm*E) have peroxisome proliferator-activated receptor (PPARγ) agonists approximately half of rosiglitazone on diabetic *db/db* mice. The mechanism was supposed by increasing absorption and the use of glucose in the peripheral cell membrane of insulin gen forming and translocating GLUT (glukosa-transporter) of swietenin activated.

The effect of a methanol extract of the seeds (MEMS) of *Swietenia macrophylla* King was evaluated to streptozotocin-induced (STZ) diabetic rats with oral glucose tolerance (OGTT) and normo-glycemic activities test. The MEMS was found to be a potential antidiabetic compared to glibenclamide. The results observed reduction on BGL, serum lipids and increasing of liver glycogen level of diabetic rats significantly. Another result observed was the lowering of fasting BGL in normal rats, group of treated extract (300 mg/kg) and glibenclamide group. Improvement of body weight profile was also observed in extract-treated diabetic rats. The research concluded the MEMS had reduction of oxidative stress associated to hypoglycemic as well as hypolipidemic effect (Maiti et al., 2007; Maiti et al., 2008; Maiti et al., 2009a, b).

The antidiabetic and antioxidant activity of the methanol extract of *S. mahagoni* bark at doses 25 and 50 mg/kg body weight (MEMB) to STZ rats to glibenclamide was evaluated. The results were shown that MEMB reduced significantly BGL and restored the body weight compared to normal rats. Other parameters were observed the decreased of TBARS, but increased of the GSH levels and CAT activities; which indicated of antioxidant activities. These would reduce free radical formation in the liver and kidney tissues of diabetic rats. These findings provoke the hypoglycemic and antioxidant activity of the MEMB extract in diabetic rats (Panda et al., 2010).

Kurniawati (2010) was tested of 1574.9 mg/kg body weight EEMS (ethanolic extract of Mahagony seed) to alloxan induced diabetic rat. The decreasing of BGL observed better compare to glibenclamida. It concluded the activities was supposed of flavonoid and saponin content of mahagony's seed extract. Al-Hasan et al. (2011) also observed *in vivo* different method of EEMS at 1000 mg/kg orally. It found no significant of the BGL treatment group compared to positive control group; but a significant reduction compared to diabetic control. The histological examination of pancreas showed destruction, retaining of islets and few de-granulations of beta cells of pancreas. These observations and results provide information that EEMS has hypoglycemic effect in experimentally induced diabetic rats.

Antidiabetic, antioxidative and antihyperlipidemic activities of 60% methanolic of mahagony seed studied at 250 mg/kg body weight to STZ rats. The seed extract was given for 21 days. The lowered of BGL as well as the glycogen level in liver observed; while improving of antioxidant enzymes (catalase/cat, peroxidase/perox) and radicals (conjugated diene/CD and thiobarbituric acid/TBAR) level in liver, kidney and skeletal muscles were obtained. The extract was also improving serum urea, uric acid, creatinine, cholesterol, triglyceride and lipoproteins level. The results indicated the MEMS potential use for the diabetes therapy, oxidative stress and hyperlipidemia related complications (De, *et al.*, 2011). Another observation was made by Dutta et al. (2014) on increasing of plasma H_2_S (hydrogen sulfide) level as H_2_S synthesis activity in plasma of water extract of *S. macrophylla* to lowering FBG in streptozotocin induced diabetic rats. H_2_S plasma functions as a neuro-modulator and a neuro-protectant against diabetes related oxidative stress.

Bera et al. (2012) found *in vivo* effects of MEMS (60% methanolic, at the 250 mg/kg body weight) to STZ rats compare to metformin. The extract and metformin were administered orally once a day. The results showed a significant reduction in the activities of hepatic hexokinase and glucose-6-phosphate dehydrogenase sequence to elevation in glucose-6-phosphatase were noted in diabetes animals compare to control animals. Level of fasting blood glucose (FBG) was elevated in diabetes animals. Activities of CAT, PEROX and SOD were diminished significantly with the elevation in TBAR. This result suggested diabetic mechanism action of the MEMS is the regeneration of acini and islets cells of the pancreas that are damaged by STZ. The extract constituents sensitized and/regenerated the ß cells that increasing insulin level serum and rectifying glycated Hb level, regulating carbohydrate enzymes metabolic activities and FBG level, respectively. Furthermore no general toxic effect was observed by SGOT, SGPT and histology to body weight of the extract-treated diabetic group. Furthermore, La Basy et al. (2015) showed EEMS improving renal dysfunction of streptozotocin-induced diabetic rats at a dose of 50, 100 and 200 mg/kg body weight for 21 days.

Suryani et al. (2013) treated the MLD-STZ rat with EEMS. It found that 250 mg/kg body weigh dose approached to control effect. It increased insulin level; decrease of TNF-⍺ and regenerate pancreatic islet. Noormalasari (2015) found that ethanolic extract of Mahagony seed would decrease BGL (347–179 mg/dl), decrease food intake and inhibit damage rate of Langerhans island and β cell of the pancreas.

Kalaivanan and Pugalendi (2011) observed of EESM (at doses 50, 100, and 200 mg/kg BW) and glibenclamide on streptozotocin-diabetic rats. They found increasing of hemoglobin (Hb) level and decreasing of glycosylated Hb level. Thus pancreas histological and biochemical finding supported insulin secretion mechanism.

Hashim et al. (2013) was also test of antidiabetic effect from various solvents of *S. macrophylla* seeds to normoglycaemic and STZ rats. It was used petroleum ether (PE), chloroform (CE) and methanol (ME). The results were shown none of the extracts had a significant effect on the BGL of 60 randomly selected normoglycaemic and diabetic rats. PE extract (at doses 500 mg/kg and 1000 mg/kg), however, significantly reduced BGL in 30 randomly selected normoglycaemic rats with IPGTT (intraperitoneal glucose tolerance tests) 30–120 min after glucose administration. PE significantly increased glucose uptake on abdominal muscle with or without insulin presence. GC-MS analysis of phytochemical content indicated terpens of diterpenes, triterpenoids and phytosterols (fucosterol and *β*-sitosterol), fatty acid methyl esters and aldehydes might act as principle compounds for the hypoglycemic effect of PE extract. Maiti et al. (2009a, b) isolated swietenine, a tetranortriterpenoids from chloroform fraction of hydroalcohol extract of *S. macrophylla* seeds, whose antidiabetic comparable to that of human insulin (p < 0.01).

The potent of aqueous extract of *S. mahagoni* leaf (AEML) as anti-diabetic was tested by oral administration to diabetic rats at 500 mg/kg for 45 days. The extract lowered the fasting BGL. There was also observation of an improvement in antioxidant glutathione components, decreasing activity of liver enzymes in the serum and reduction in body mass loss in treated groups. Thus, it can be proposed that the effect may be mediated through increasing the antioxidant strength, improving liver glycogen content, balancing the lipid components in serum, decreasing the muscle protein catabolism and improved overall health (Naveen and Urooj, 2015).

Hypoglycemic activity was also observed from ethanolic dried extract of *S. mahagoni* (500 mg and 1000 mg/kg body weigh) to alloxan-induced mice much greater compare to glibenclamide (Wardani, 2016). Alloxan and streptozotocin chemicals are usually used for the induction of diabetes mellitus *in vivo* experimental, since both are destructive to Langerhans *ß* cells of the islets. Histopathological observation on the restoring of the density and percentage of *ß* cells diabetic extract treated might indicated the regeneration. Some plant extracts observed summation to regenerate of *ß* cells, restore insulin secretion from surviving *ß* cells of the islets of Langerhans and lowering of BGL (Szkdelski, 2001; Singh and Gupta, 2001; Yadav et al., 2008). The water infusion and methanol extracts of mahogany bark (Falah et al., 2010) exerted regenerating of *ß* cells, restoring insulin of the cell (pancreatrophic) producing or the extract may have insulin like substances with dose-dependent manner (Adewole and Ojewole, 2007).

The result of *in vivo* antidiabetic of EEMS to alloxan intraperitoneal administration to mice; showed dose-independent manner of antidiabetic activity compare to glibenclamide. This research supported the developing of mahagoni seed extract to pharmaceutical formulation, since the mahagoni extract used in dried formulation (filling with avicel: cab-o-sil by ratio of 70:30 and dried 4:1) (Sukardiman et al., 2017).

Limited clinical study of *S. mahagoni* was observed in 68 type II diabetic patients with pre- and post-test control group design experimental. Result was shown that 85.3% treatment group has glucose blood level 90–199 mg/dl. Bivariate analysis result was shown the potential of *Mahagoni* seed to reducing BGL compare to glimepiride (Astuti et al., 2017).

Sukardiman et al. (2013) tested the combination of herbal tea of *Andrographis paniculata* herbs and *S. mahagoni* seeds (2:1), which has given for seven days at the dose of 0.4 ml/20g body weight; resulted the highest BGL reduction (88.20 ± 43.16 mg/dl) of alloxan diabetic mice compare to other ratios.

Govindappa (2007) reviewed 419 plant species of 133 families of plant extracts or phytochemicals to *in-vitro* and *in-vivo* of antidiabetes therapy. It resumed that the plant extracts involved different mechanisms in diabetes. Antidiabetic molecules from different parts of the plant extracts produced signal transduction in restoring insulin production or normalize BGL. The *Azadirachta indica* A. Juss and *Trichilia emetica* of Meliaceae's family, were include on the list, but none of Mahagony*.* The review also suggested the antidiabetes mechanism of the phytochemicals. Azadirachtin and nimbin (bioactive compound of *A. indica* seed) are supposed increased peripheral glucose uptake by inhibiting of epinephrine on glucose metabolism (Donga et al., 2011) while nimbidin, nimbin, nimbidol and nimbosterol (leaves extracts content) have glycogenolytic of epinephrine blocked (Chattopadhyay, 2007). Both extracts increased insulin secretion (Tripathi et al., 2007). The flavonoid-rich fractions of *T. emetica* extract was shown antidiabetic, anti-lipidemic and antihypertensive activities (Konaté; et al., 2014). Mendes and Bogle (2015) was also proposed mechanism of alkaloids, saponins, vitamins, polyphenols, flavonoids and limonoids as hypoglycemic bioactive component. Limonoids content of the hexane extract of *S. humilis* also noted as hypoglycemic, reducing serum triglycerides and uric acid. This result suggested the insulin sensitizing mechanism and glycogen synthesis activating, abdominal fat rats eliminating, blood triglycerides eliminating and increasing adipose tissue glucose uptake (Magallanes et al., 2015).

Swietenine, a limonoid from *S. macrophylla*, have moderate hypoglycemic and reduce triglycerides in blood diabetic rat model (Dewanjee et al., 2009; Maiti et al., 2009a, b). Antidiabetic synergistic mechanism and hypolipidemic activity on different molecular targets was also proposed (Mendes and Bogle, 2015) (Table 3). A mixture of many components in the extract of the plant would enhance the bioavailability of one or several compounds of the extract, thus improving its pharmacological action. Synergistic effect made difficult to prove mechanism and might produce contrary result in some research. Medicinal chemistry-based analysis of the phytochemical can promote the prospects as natural products in managing diabetes.

**Table 3:** Proposed synergistically mechanism of antidiabetic and hypolipidemic of phytochemicals class.

Molecular Target	Compound Class	Mechanisms
Enzyme (α-glucosidase, α-amylase) inhibition activity	Flavonoids, sterols, terpens	Inhibition of glycogen phosphorylases and glucose 6-phospatase, inhibit lipid peroxidation, stimulate insulin secretion, protective regeneration cell β function
Acetyl CoA activation	Cathechin	Inhibit lipid peroxidation, stimulate insulin secretion, protective regeneration cell β function, activate of AMPK, increase expression level of GLUT4 transporter
α-glucosidase inhibition activity	Saponins	Decrease insulin resistance and inhibits carbohydrates absorption, increase expression level of GLUT4 transporter, inhibition of glycogen phosphorylases and glucose 6-phospatase
Inhibition of mitochondrial function/stimulating of glycolysis	Berberine	Inhibit lipid peroxidation, stimulate insulin secretion, protective regeneration cell β function, activate of AMPK
Insulin sensitizising, α-amylase inhibition	Limonoids	–

Molecular targeting approached of antidiabetic mechanisms were proposed by some researchers. Molecular target on SGLT2 (Vigneshwaran and Lalitha, 2016), peroxisome proliferator-activated receptor gamma (PPARɤ) (Jian et al., 2018), and α-amylase inhibitor (Ponnusamy et al., 2010) have been done. These studies were exploring the potency of hypoglycemic phytochemical, or improving the activity of provided antidiabetic substances on its specific target. Zapata-Sudo et al. (2012) and Jian et al. (2018) examined the N-benzylbenzamide or sulphonylhydrazone antidiabetic derivatives on PPARɤ agonist; while Ponnusamy et al. (2010) was working with gedunin and azadiradione limonoids of *A. indica* on α-amylase inhibitor, so did Vigneshwaran and Lalitha (2016) with antidiabetic phytocehmical of the *Swietenia mahagoni* seeds. Jian et al. (2018) found the possible binding active site of PPARɤ on the residues of His323, Tyr473, Ser289 and Ser342 with the hydrogen bond interactions. Zapata-Sudo et al. (2012) was found LASSBio-1471, a novel sulfonylhydrazone derivative ligand, have least theoretical energy binding for PPARɤ among all new compounds. It was effective on reducing BGL from 548.4 ± 26.0 to 259.6 ± 73.1 mg/dL and paw withdrawal from 21.9 ± 1.7 to 36.7 ± 1.2 g of STZ-diabetic rats’ neuropathy (20 mg/kg, i. p.) for 7 days. The structure of LASSBio-1471 was of 1,3-benzodioxole subunit which inhibited and/or induced CYP450. Furthermore, Vigneshwaran and Lalitha (2016) were analyzed of *S. mahagoni* seed 18 compounds and dapaglifozin reference antidiabetic drug to SGLT2 targeted molecule. The content included Mahonin, Sweitenin B–F, Secomahoganin, Swietenollide, Swietemahonin A-G, Swietemahonolide, and oleanolic acid. It was found that all compounds have lesser, and oleanolic acid has the least binding energy compare to the synthetic reference dapaglifozin (-7.77 kcal/mol). This new approach not only provided supportive information for empirical or scientific factual, but also offered effective screening methods, as versatile tools for antidiabetic natural substances drug discovery experiments.

The toxicity of the seed and ethanolic extract of *Swietenia macrophylla* and *Swietenia mahagoni* were tested with *in vitro* and *in vivo*. The result was obtained that *S macrophylla* seed orally given of 2 g/kg BW is safe to rats (not influenced either by food or water intake, weight and histology of vital organ, the biochemical and hematological parameters as well); neither observed of toxicity signs nor deaths during the toxicity study period (Balijepalli et al., 2015). Furthermore, the toxicity of *S. mahagoni* seed extract showed safe to mild toxic range. Brine shrimp lethality *in vitro* test resulted LD_50_ of methanolic extract *S. mahagoni* Jacq. seed (MEMS) is more than 2500 mg/kg; which is classified as a relatively nontoxic (Sahgal et al., 2010). This result was also supported by Ghosh et al. (2009) which found increasing dose of intraperitoneal injection of *S. mahagoni* seed extract to mice was non-toxic (up to 1.2 g/kg body weigh up to a day). Tough *in vivo* acute toxicity test of *mahagoni* ethanolic extract (LD_50_ 7.998 g/kg body weigh) was observed the changes on the kidney and liver histology; thus categorized as mild toxic of (Saputri, 2014).

## 6 Conclusion

*S. mahogani* is one of among three plants of *Swietenia*'s Meliaceae family, which has more potential to develop as antidiabetic phytomedicine agent. Its seed/bark or leaves of ethanolic/methanolic/aqueous/petroleum/hexane extracts have shown antidiabetes activities by reducing BGL, restoring liver and ß-cells islet function, blocking epinephrine function, inhibiting of α-amylase and β-glucosidase, antioxidant and antihiperlipidemia mechanisms. Phytochemical compounds of *S. mahogani* consist of the phenolic (flavonoids (swietemacrophyllanin, catechin and epichatechin) and tannins), triterpenoids, tetranortriterpenoid (limonoid: mahonin, secomahoganin, swietmanins, swiemahogins, swietenine and swietenolide), saponins and alkaloids which are known as antidiabetic bioactive principles. To use it as an antidiabetic further, more extensive clinical trials and biomarkers of active compounds determination are required.

## Declarations

### Author contribution statement

All authors listed have significantly contributed to the development and the writing of this article.

### Funding statement

This work was supported by Universitas Airlangga Grant for Article Review Program 2019

### Competing Interest Statement

The authors declare no conflict of interest.

### Additional Information

No additional information is available for this paper.
